# Does testosterone predict women’s preference for facial masculinity?

**DOI:** 10.1371/journal.pone.0210636

**Published:** 2019-02-27

**Authors:** Urszula M. Marcinkowska, Samuli Helle, Benedict C. Jones, Grazyna Jasienska

**Affiliations:** 1 Department of Environmental Health, Faculty of Health Sciences, Jagiellonian University Medical College, Cracow, Poland; 2 Section of Ecology, Department of Biology, University of Turku, Turku, Finland; 3 Institute of Neuroscience and Psychology, University of Glasgow, Glasgow, United Kingdom; Tilburg University, NETHERLANDS

## Abstract

The influence of sex hormones on women’s mate preferences has been an intensively discussed topic for more than a decade. Yet the extent to which levels of sex hormones, and testosterone in particular, influence women’s mate preferences is unclear. Thus, the current study used multilevel modelling to investigate putative relationships between salivary testosterone and facial masculinity preferences in a sample of 68 women, while controlling for their age, partnership status, and sociosexuality. We found no significant associations between masculinity preferences and either individual differences or within-woman changes in testosterone. We did find however, that sociosexuality was positively correlated with masculinity preferences. Although it has previously been suggested that testosterone is related to women’s facial masculinity preference, our data do not support this proposal.

## Introduction

It has been hypothesized that women’s preferences for putative partner characteristics change throughout the menstrual cycle depending on the probability of current conception and, thus, on sex hormonal status. “The ovulatory shift” hypothesis proposes that women prefer partners with “good genes” (i.e., genes that are beneficial for their offspring [1]) around ovulation while directing their attention towards potential partners providing high paternal investment and parenting skills at less fertile points of the cycle (e.g., during late luteal phase). Therefore, we would expect women to express increased masculinity preferences around ovulation when fertile and decreased masculinity preference in non-fertile phases [2]. As women’s fertility is directly related to their hormonal status, it is a straightforward prediction that such fluctuation of preferences should be related to hormonal changes that occur over the menstrual cycle.

Levels of sex hormones are not constant throughout life. For example, they change due to current fertility status (depending on the position in the menstrual cycle) and life-long fertility (being highest during peak reproductive years). Testosterone (T) is thought to be the highest around ovulation [3], although some studies showed that changes in testosterone levels across the menstrual cycle are smaller than circadian changes [4].

Regardless of the significant variation in circadian T levels, some studies have reported relationships between masculinity preferences and daily, but not the average, T levels [5, 6]. Another study, examining preference towards vocal masculinity, found no relationship between masculinity preferences and T levels [7]. However, in this latter study, T levels were not measured directly, but were assigned, based on previously published data (i.e., actuarial tables). Based on hormones measured from saliva of the participants, Pisanski et al. [8] found that changes in estradiol, but not testosterone predicted increased vocal masculinity preferences. In two other published studies, Roney at al. [9, 10] found that women’s preference for facial cues to high testosterone were not correlated with their T levels. A more recent study by Ditzen et al. [11] found that estradiol, but not T, modulated masculinity preferences, with high estradiol being related to stronger masculinity preference. However, this association was supressed when women’s cortisol was high (i.e., their stress level was high). Welling et al. [5] found that testosterone levels predicted women’s preference towards facial masculinity when comparing preferences at two points in the menstrual cycle, but did not directly relate preferences to measured hormone levels. Bobst et al. [6] found a positive relationship between salivary testosterone levels and masculinity preference when women were tested during the early follicular phase. Interestingly, the reward value of sexually dimorphic faces presented to naturally cycling women was greater when women had high T levels [12]. This suggests that these women found sexual dimorphism more rewarding depending on levels of sex hormones, but the effect was visible only for attractive faces and feminine female faces and not for masculine male faces.

None of the published studies so far have included variables potentially confounding relationships between T and women’s preferences in the analyses, such as age, relationship status, and sociosexuality. It has been shown that these characteristics influence women’s attraction towards masculine men [13–15] and it could be argued that their influences should be controlled for when examining the relationship between sex hormones and women’s preferences. Moreover, not all published studies verified sexual orientation of participants, and, more importantly, none tracked full hormonal profiles across the menstrual cycle.

In this study, we investigated the relationships between levels of testosterone, both on the same day that the preferences were measured and when averaged over 15 days during the whole cycle, and women’s preferences for male facial masculinity. In other words, we designed a study that allowed both intra- and inter-participant analyses, had a relatively large sample size of 68 women, and directly verified occurrence of ovulation in a cycle.

## Methods

This research has been approved by the Bioethics Committee of the Jagiellonian University Medical College, under the agreement number KBET/250/B/2014 and all clinical investigations have been conducted according to the principles expressed in the Declaration of Helsinki. Written and informed consent has been collected from all participants upon agreeing for participation in the study.

Women were recruited from the Malopolska region of Poland. Criteria for inclusion in the study were: having regular menstrual cycles (such that a difference between consecutive cycles lengths was usually not larger than ±5 days), not having diabetes, no medically diagnosed health problems of the reproductive system, and not being pregnant, breastfeeding or taking hormonal contraception for at least 3 months prior to participation. Out of the 110 women recruited, 102 completed the study. Eight women scored 4 or higher on the Kinsey Sexual Orientation Scale (self-defined themselves as bi- or homosexual [16]) and were, therefore, excluded from analysis, as sexual orientation can influence preferences towards facial features [17]. Additional 26 took preference tests during non-ovulatory cycle (verified based on LH tests). Raw, anonymized data can be found in S2 File.

Based on the same dataset, two articles have been already published [18], [19]. In Marcinkowska et al. [19] levels of two other ovarian sex hormones were investigated, progesterone and estradiol. Due to obtaining funding for testosterone measurements after the publication of the mentioned results analyses that used testosterone measurements could not have been added to previous articles. In Marcinkowska et al. (2018) none of the reported daily measurements of the two sex hormones were significantly related to masculinity preferences [19]. The second article published [18] reported changes in women’s preferences for masculinity throughout the menstrual cycle depending on relationship status, self-judged attractiveness and sociosexuality. The current article focuses on testosterone, another sex hormone that possibly influences sexual preferences in women. After obtaining null results for two other sex hormones in previous studies we decided to examine whether testosterone might be at least partly responsible for masculinity preferences changes. The analyses involving testosterone were conducted after the previous articles have been published, but we believe that these articles together provide a more coherent picture of hormonal bases of sexual preferences, particularly given the mixed results for T and masculinity preferences that have been reported in the literature (see Introduction).

### Procedure

During the introductory meeting, participants were instructed how to collect and store saliva samples, gave written instructions, and were given a set of 2 mL centrifuge tubes with minimum amount of required saliva marked. Saliva was collected daily, usually immediately after waking up, starting from the first day of menstrual bleeding until the end of the cycle (one day before next menstrual bleeding). LH urine tests were conducted from the 10th until 20th day of the cycle or until obtaining a positive result of the test (following [20]). Participants attended 3 meetings. The first meeting was arranged well before expected ovulation (early follicular phase, not later than the 10th day of the cycle, on average 23 days before the onset of next menses, SD = 2.17). The second meeting occurred around ovulation (fertile, peri-ovulatory phase, on average 14 days before the onset of next menses, SD = 4.49). The third meeting was scheduled approximately one week after ovulation (during luteal phase, on average 4 days before the onset of next menses SD = 3.68). It was not possible to facilitate a luteal phase meeting before the onset of the next menses due to premature arrival of the next menses for 12 cycles. For this subsample, the third meeting took place on the 1st or 2nd day of the next menses, as this time of the cycle is also a low-fertility period [21].

**Fig 1:**
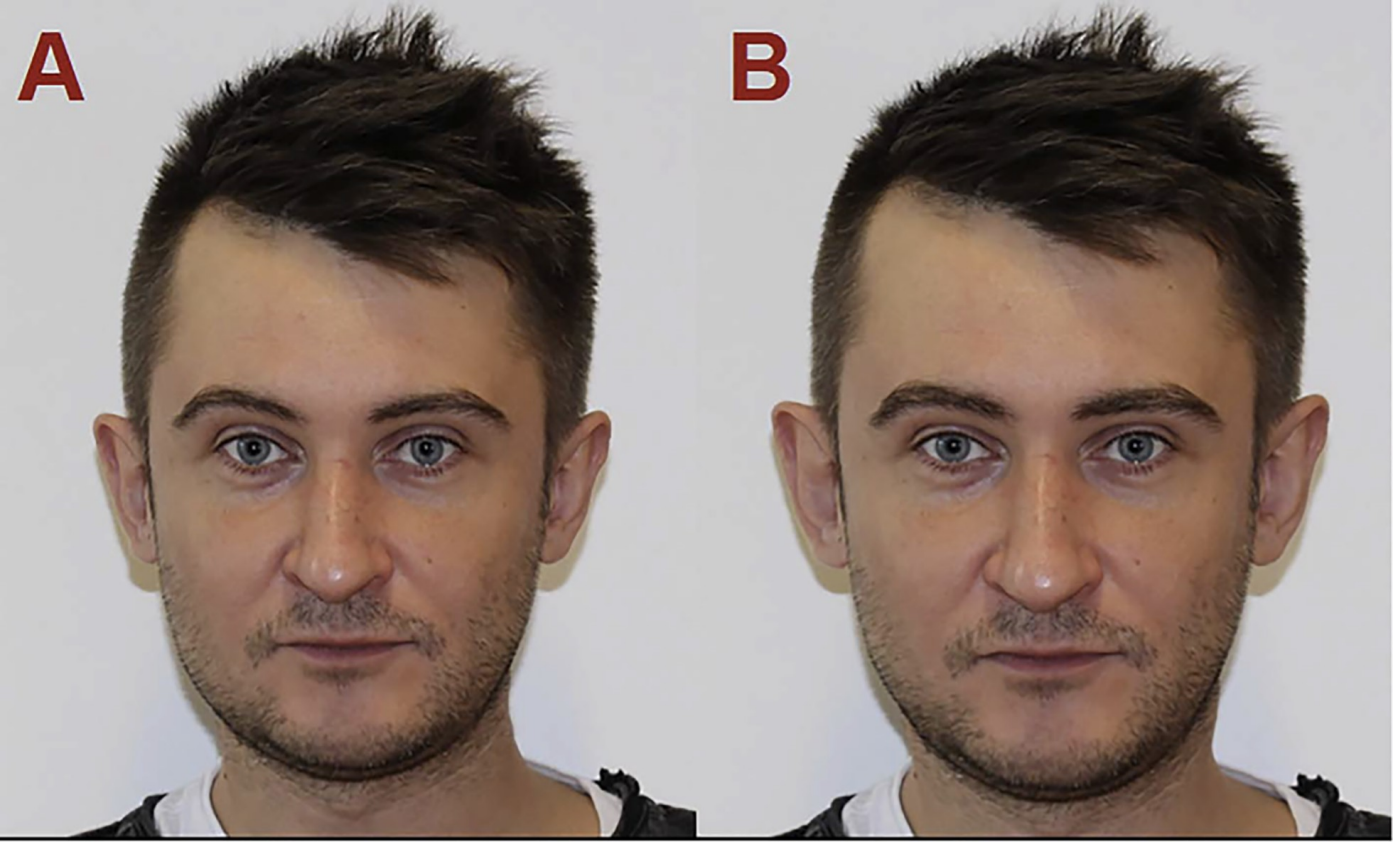
Example of the visual stimuli (A: Feminized version of the face, B: Masculinized version of the face).

During each of the three meetings, participants completed three surveys: the attractiveness judgement of male faces that differ in sexual dimorphism, the socio-demographic questionnaire (e.g. age, previous use of hormonal contraception, date of onset of last menses, menses regularity and average length of the cycle, sexual orientation and relationship status), and the entire Sociosexual Inventory Revised (SOI-R [22], for description of participant’s responses see: Table 1). Further, via 2-alternative forced-choice participants selected the more attractive of the two simultaneously presented facial photographs. All visual stimuli were obtained using PSYCHOMORPH software [23], thus it was possible to manipulate only a selected set of cues: male faces differed only in sexual dimorphism, while all other characteristics (e.g. skin texture, symmetry) were held constant. Each slide depicted two versions of the same facial photograph (i.e., one manipulated to be 50% more similar to the average age-matched female face and one 50% more similar to the average age matched masculine face, Fig 1). The individual pictured in Fig 1 has given written informed consent (as outlined in PLOS consent form) to publish this image. Pictures were presented in a random order.

**Table 1:** Description of participants.

	n	Mean	Min	Max	SD
Age (years)	68	29.01	20.67	37.50	4.67
Testosterone levels during follicular phase (pg /ml)	62	26.15	6.25	57.90	11.69
Testosterone levels during peri-ovulatory phase (pg /ml)	66	28.20	2.46	70.71	12.94
Testosterone levels during luteal phase (pg /ml)	60	23.52	1.25	67.84	11.12
Average testosterone levels throughout the cycle (pg/ml)	66	26.68	6.05	51.86	9.68
Masculinity preference during follicular phase (%)	67	0.30	0.00	0.88	0.25
Masculinity preference during peri-ovulatory phase (%)	67	0.38	0.00	1.00	0.32
Masculinity preference during luteal phase (%)	67	0.33	0.00	1.00	0.25
Sociosexuality Inventory Score	68	3.09	1.04	6.41	1.45

### Hormonal assays

Levels of testosterone (T) were measured in daily collected saliva samples. Participants were instructed to collected saliva samples immediately after waking up and at least 30 minutes after eating, drinking or smoking, and to freeze a sample immediately upon collection. Hormonal measurements were conducted using commercially available hormonal assays of DRG International Incl. Elisa plates SLV3013 for testosterone (sensitivity: 1.9 pg/ml, standard range: 10–5000 pg/ml). To obtain highest standard of the measurements, all hormonal assays were conducted in duplicates and the quality of hormonal measurements was controlled for each plate separately by including (also in duplicates) samples of known concentrations (“pools”) of T (in total these control measurements consisted of, on average 18 pools per plate). Inter- and intra-assay coefficients of variability (CVs) were computed and were on acceptable levels: inter-assay CV was 12.30%, and intra-assay was 1.27%, [24].

### Statistical methods

Generalized linear multilevel modelling [25] was used to examine whether testosterone levels during the different periods of the cycle or averaged over the whole cycle were associated with women’s preference for masculinity due to 1) non-normal response variable 2) missing data and thus use of multiple imputation and 3) group-centering to test the hypothesis of main interest. Owing to some missing data in independent variables (see Table 1), we started by imputing 20 data sets using multilevel sequential regression (or chained equations) approach using unrestricted model with Bayesian estimator. Our final results are thus based on the combined results from these 20 data sets. The outcome variable, women’s preference for men’s facial masculinity, was modelled as the rate of positive choices (i.e., counts) for masculinity out of the total of eight trials, which was used as an offset variable since few women did not complete all the trials. No evidence for overdispersion was found (dispersion parameter = 0.00, *p* = 0.99), and thus Poisson error distribution and log link function were used. Change in women’s preference for masculinity during the three time periods of menstrual cycle (i.e., during follicular, peri-ovulatory and at luteal phase) was modelled as a bilinear spline model, because we expected a non-linear change in preferences. We used time of ovulation as the knot point that separated the two linear regression slopes (i.e., one from follicular phase to ovulation and one from ovulation to luteal phase), which also centered the intercept at the time of ovulation [26]. Both the linear slopes and the intercept were further modelled as random (variance) factors in order to allow for between-subject variation in those quantities [21]. Levels of testosterone during follicular phase, peri-ovulatory and luteal phase were group-mean centered (i.e., the values were subtracted from a within-woman mean value), since this model specification answers our main study question [27]: whether the ovulation phase-specific measure of testosterone or its average value during the whole cycle were more closely associated with women’s preference for masculinity. This question was statistically tested by comparing within- and between-women regression parameters of testosterone [27]. Hence, as within-women predictors, the model included testosterone measured at the three different phases of the menstrual cycle and the two variables describing bilinear change in masculinity preferences [26]. The estimated mean value of testosterone during the whole cycle, based on 15 consecutive daily measurements, was used as the between-women predictor. Women’s age, their relationship status and socio-sexual index were also modelled as between-women covariates and were thus all grand-mean centered [27]. To facilitate model convergence, the value of testosterone was divided by 100. Bilinear spline indicators were also divided by 10 in order to increase the numeric precision of their variance component. The model was estimated for each multiple imputed data set using robust maximum likelihood, after which the results were compiled. As testosterone might be related to or work in accordance with other two sex hormones, namely estradiol and progesterone, the model then was applied including two additional sex hormones. All analyses were conducted using Mplus 8 [28], exact formula used can be found in S1 File.

## Results

For descriptive statistics on testosterone values see Table 1, (for progesterone and estradiol see Table A in S1 File).

Statistical model showed that at both the within- and between-women levels (i.e., during the follicular, peri-ovulatory, and at the luteal phase and averaged over the whole cycle, respectively), the concentrations of testosterone were not associated with women’s preference for masculinity (Table 2). In other words, testosterone levels measured at the different phases of the cycle or averaged over the whole cycle were not statistically associated with their preference for masculinity. Furthermore, we found no statistical evidence suggesting that the influence of testosterone level on masculinity preference differed between within- and between-women levels (i.e., the parameter “Testosterone_cm—cmc_”in Table 2). Of the covariates included in this model, only socio-sexuality index was associated with masculinity preference, as 1-unit increase in socio-sexual index increased the preference for masculinity by 18.7% (95% CIs: 6.5%, 32.1%).

**Table 2:** The combined results of generalized linear multilevel models. The results averaged over 20 imputed data sets, examining the influence of testosterone concentration during the three phases of a menstrual cycle and overall average of the whole cycle on women’s preference for masculinity at the within- and between-women levels (n = 68 women, 204 observations). Note that all parameters are on log scale. Suffix “CMC” denotes to variables with cluster-mean centering at the within-women level and suffix “CM” denotes to variables with cluster means at the between-women level.

	Estimate	S. E.	z-value	p
**Within level**				
Testosterone_cmc_	0.249	0.512	0.486	0.627
Offset	1			
				
**Between level**				
Intercept	-1.183	0.120	-9.883	<0.0001
Slope_follicular phase–peri-ovulaory_	2.287	0.866	2.642	0.008
Slope_peri-ovulatory—luteal phase_	-1.242	0.851	-1.460	0.144
Testosterone_cm_	0.955	0.938	1.019	0.308
Age	-0.013	0.021	-0.599	0.549
Relationship status	-0.303	0.188	-1.612	0.107
Socio-sexuality index	0.171	0.055	3.109	0.002
				
**Variance terms**				
Intercept	0.335	0.111	3.029	0.002
Slope_follicular phase—ovulation_	0.042	0.025	1.683	0.046
Slope_ovulation—luteal phase_	0.026	0.014	1.925	0.027
				
**Parameter contrasts**				
Testosterone_cm—cmc_	0.707	1.077	0.656	0.512

The same model simultaneously including also progesterone and estradiol did not change the above conclusions with respect to the associations of testosterone on masculinity preferences (all results for extended model are reported in Table B in Supplementary Information S1 File). To account for possible misclassification of participants as “peri-ovulatory” even when they attended second meeting later than 24 hours after the positive rest of LH test we have conducted two additional analyses including 1) only participants who attended the peri-ovulatory meeting not later than 24 hours after the recorded positive LH test results (N = 43) and 2) only participants who attend second meeting not more than 48 hours later (N = 54). None of the additional analyses based on the narrowed samples yielded a statistically significant result between testosterone concentrations and masculinity preferences (Table C and Table D in S1 File).

Women’s preference for masculinity increased from follicular phase to ovulation, on average, by 25.7% (95% Confidence intervals (CIs): 6.1%, 48.9%; Table 2). After that, preference for masculinity decreased, on average, by 11.7% (95% CIs: -25.3%, 4.4%) from ovulation to luteal phase, although this change was not statistically significant (Table 2). We also found evidence suggesting that women differed in their pattern of change with respect to their preference for masculinity during the cycle. This was indicated by significant variance components for the linear changes from follicular phase to ovulation, and from ovulation to luteal phase (Table 2). Moreover, a significant variance component for random intercept showed that women differed in their preference for masculinity during ovulation (Table 2).

## Discussion

Our study showed no evidence that preferences for masculine men faces tracked within-woman changes in testosterone levels during menstrual cycle or were related to between-women differences in average testosterone levels during the whole cycle. Thus, our results do not support the hypothesis that women show stronger preferences for masculine men on days of the menstrual cycle where testosterone is high [5] or that women with higher basal testosterone levels show stronger preferences for masculine men [6]. Moreover, even after including two other sex hormones in the analyses, we did not find any significant relations between current hormonal level and preferences. Our null results for testosterone and masculinity preferences are, however, consistent with other recent studies that have reported no significant relationships between testosterone and women’s masculinity preferences [11, 29]. While testosterone may contribute to women’s general sexual desire ([30] but see also [31]), our null results add to a growing body of evidence suggesting that testosterone does not play an important role in women’s masculinity preferences. This result coupled with the pool of mixed results of published studies underlines that the possible changes of women’s preferences throughout the cycle are not a straightforward result of the fluctuations of testosterone, but are far more complex than previously assumed [32].

Interestingly we did find a difference in women’s preferences between follicular and peri-ovulatory meetings; women judged masculinised faces as attractive more often during peri-ovulatory meeting (in contrast to results reported in [33]). This result could partially support the peri-ovulatory shift hypothesis, although there are some important hindrances. Following the shift hypothesis, masculinity preference should mirror the fluctuations in fertility. If that was the case, we should observe strongest drop in preference in luteal phase (statistically not significant in this study). Further, in our study a significant intra-individual variation was observed in the pattern of masculinity preference shifts as indicated by statistically significant variance terms of the preference slopes. These results support the idea (as suggested in previously published article [18]) that around-ovulatory shifts would only be in place for a certain groups of women, i.e. women who are not in a relationship or have high self-judged attractiveness. However, more studies are needed to disentangle this inherently complex phenomenon.

Another possible reason why we did not find a significant relationship between testosterone and preferences is that we examined facial masculinity only. It is possible that facial masculinity is not a sufficiently strong (or valid) signal of good genes in men [34, 35] and that preference for body, vocal or behavioral masculinity in men would be more related to women’s testosterone levels. Our conclusions based on available data are hence limited to the facial cues.

Although we found no evidence that masculinity preferences were related to women’s testosterone levels, we did find that women who reported greater openness to short-term, uncommitted sexual relationships (as indexed by responses on the revised Sociosexual Orientation Inventory) had stronger preferences for masculine men. This result is consistent with previous studies finding that women show stronger masculinity preferences when assessing men’s attractiveness for hypothetical short-term, as compared with long-term, relationships (e.g. [15]) and reporting similar correlations between sociosexual orientation and masculinity preferences [36]. Such results could emerge because the antisocial personality traits typically ascribed to masculine men (e.g., emotional coldness,[37]) are less important in the context of short-term relationships or reflect women seeking short-term relationships favouring men they perceive as being less interested in long-term relationships. While these two possibilities could be a useful avenue for future research, we note here that not all studies have reported correlations between sociosexuality and women’s masculinity preferences [38].

In summary, our results compliment previously published studies on relation between sex hormones and preferences, and show no evidence that women’s masculinity preferences are related to their testosterone levels during menstrual cycle. Thus, our null results add to a growing body of evidence suggesting that previously reported links between women’s masculinity preferences and testosterone are not robust.

## Supporting information

S1 File (DOCX)

S2 File (CSV)
