# Supplementary material for: Does testosterone predict women’s preference for facial masculinity?
Source: PLoS One. 2019 Feb 27;14(2):e0210636. doi: 10.1371/journal.pone.0210636 (PMC6392222; doi:10.1371/journal.pone.0210636)
Supplement: S1 File — (DOCX) [file pone.0210636.s001.docx]

**Electronic Supplementary Material 1**

The formula of the generalized linear multilevel model fitted (using variable labels instead of

algebraic symbols) was:

masculinity preference ij = γ 00 + γ 10 *min(i- k i ,0) ij + γ 20 *max(i- k i ,0) ij + γ 30 *testosterone ij +1*offset ij + γ 01 *testosterone j + γ 02 *age j + γ 03 *relationship status j +

γ 04 *socio-sexual index j + μ 0j + μ 1j *min(i- k i ,0) ij + μ 2j *max(i- k i ,0) ij

where the subscripts i and j denote to facial masculinity preference for a woman j at the

measurement occasion i, respectively. Note that k i denotes to a knot point (i.e., ovulation) at

which point the intercept (γ 00 ) was centered.

**Table A. Mean levels of previously measured progesterone and estradiol (SD provided in brackets, [1]).**

|  |  | **Follicular Phase** | **Around Ovulation** | **Luteal**  **Phase** |
| --- | --- | --- | --- | --- |
|  | |  |  |  |
|  | Progesterone levels (pg/ml) | 53.12 (54.56) | 88.02 (79.11) | 109.4 (86.86) |
|  | Estradiol levels (pg/ml) | 6.96 (7.53) | 8.86 (8.5) | 7.06 (6.23) |

Inter- and intra-assay coefficients of variability (CVs) were computed and were on acceptable levels: for progesterone interassay CV was 14.1%, and intra-assay was 4.9% and for E2, inter-assay

CV was 10.01%, and intra-assay was 7.5%.

**Table B.** **The combined results of generalized linear multilevel model for all participants. including three sex hormones.**

The results of generalized multilevel models, averaged over 20 imputed data sets, examining the influence of women’s sex hormones during menstrual cycle on their preference for masculinity at the within- and between-women levels (n = 68 women, 204 observations). Note that all parameters are on log scale. Suffix “CMC” denotes to variables with cluster-mean centering at the within-women level and suffix “CM” denotes to variables with cluster means at the between-women level.

|  |  | **Estimate** | **S. E.** | **z-value** | **p** |
| --- | --- | --- | --- | --- | --- |
| **Within level** | |  |  |  |  |
|  | Progesterone_cmc_ | 0.055 | 0.094 | 0.582 | 0.560 |
|  | Estradiol_cmc_ | 1.289 | 0.655 | 1.967 | 0.049 |
|  | Testosterone_cmc_ | 0.031 | 0.555 | 0.056 | 0.956 |
|  | Offset | 1 |  |  |  |
|  |  |  |  |  |  |
| **Between level** | |  |  |  |  |
|  | Intercept | -1.230 | 0.120 | -10.052 | <0.0001 |
|  | Slope_follicular phase - ovulation_ | 1.857 | 0.915 | 2.030 | 0.042 |
|  | Slope_ovulation - luteal phase_ | -1.123 | 0.870 | -1.291 | 0.197 |
|  | Progesterone_cm_ | -0.086 | 0.115 | -0.748 | 0.454 |
|  | Estradiol_cm_ | 0.222 | 1.394 | 0.159 | 0.873 |
|  | Testosterone_cm_ | 0.979 | 0.960 | 1.019 | 0.308 |
|  | Age | -0.012 | 0.021 | -0.544 | 0.587 |
|  | Relationship status | -0.302 | 0.187 | -1.618 | 0.106 |
|  | Socio-sexual index | 0.167 | 0.054 | 3.091 | 0.002 |
|  |  |  |  |  |  |
| **Variance terms** | |  |  |  |  |
|  | Intercept | 0.328 | 0.110 | 2.987 | 0.003 |
|  | Slope_follicular phase - ovulation_ | 0.032 | 0.016 | 2.046 | 0.021 |
|  | Slope_ovulation - luteal phase_ | 0.019 | 0.010 | 1.911 | 0.028 |
|  |  |  |  |  |  |
| **Parameter contrasts** | |  |  |  |  |
|  | Progesterone_cm - cmc_ | -0.141 | 0.139 | -1.010 | 0.312 |
|  | Estradiol_cm - cmc_ | -1.067 | 1.492 | -0.715 | 0.475 |
|  | Testosterone_cm - cmc_ | 0.948 | 1.122 | 0.845 | 0.398 |

**Table C**. **The combined results of generalized linear multilevel model for participants who attended 2^nd^ meeting not more than 24h after positive LH test result.**

The results averaged over 20 imputed data sets, examining the influence of testosterone concentration during the three phases of a menstrual cycle and overall average of the whole cycle on women’s preference for masculinity at the within- and between-women levels (N = 43 women, 129 observations). Note that all parameters are on log scale. Suffix “CMC” denotes to variables with cluster-mean centering at the within-women level and suffix “CM” denotes to variables with cluster means at the between-women level.

|  |  | **Estimate** | **S. E.** | **z-value** | **p** |
| --- | --- | --- | --- | --- | --- |
| **Within level** | |  |  |  |  |
|  | Testosterone_cmc_ | -0.030 | 0.684 | -0.043 | 0.966 |
|  | Offset | 1 |  |  |  |
|  |  |  |  |  |  |
| **Between level** | |  |  |  |  |
|  | Intercept | -1.376 | 0.150 | -9.165 | <0.0001 |
|  | Slope_follicular phase - ovulation_ | 2.616 | 1.200 | 2.181 | 0.029 |
|  | Slope_ovulation - luteal phase_ | -0.097 | 0.272 | -0.356 | 0.722 |
|  | Testosterone_cm_ | 0.390 | 1.287 | 0.303 | 0.762 |
|  | Age | -0.020 | 0.030 | -0.690 | 0.490 |
|  | Relationship status | -0.148 | 0.311 | -0.478 | 0.633 |
|  | Socio-sexuality index | 0.169 | 0.086 | 1.972 | 0.049 |
|  |  |  |  |  |  |
| **Variance terms** | |  |  |  |  |
|  | Intercept | 0.410 | 0.146 | 2.812 | 0.005 |
|  | Slope_follicular phase - ovulation_ | 0.085 | 0.073 | 1.160 | 0.13 |
|  | Slope_ovulation - luteal phase_ | 0.001 | 0.005 | 0.213 | 0.416 |
|  |  |  |  |  |  |
| **Parameter contrasts** | |  |  |  |  |
|  | Testosterone_cm - cmc_ | 0.419 | 1.404 | 0.299 | 0.765 |

**Table D**. **The combined results of generalized linear multilevel models for participants who attended 2^nd^ meeting not more than 48h after positive LH test result.**

The results averaged over 20 imputed data sets, examining the influence of testosterone concentration during the three phases of a menstrual cycle and overall average of the whole cycle on women’s preference for masculinity at the within- and between-women levels (N = 54 women, 162 observations). Note that all parameters are on log scale. Suffix “CMC” denotes to variables with cluster-mean centering at the within-women level and suffix “CM” denotes to variables with cluster means at the between-women level.

|  |  | **Estimate** | **S. E.** | **z-value** | **p** |
| --- | --- | --- | --- | --- | --- |
| **Within level** | |  |  |  |  |
|  | Testosterone_cmc_ | 0.112 | 0.568 | 0.197 | 0.844 |
|  | Offset | 1 |  |  |  |
|  |  |  |  |  |  |
| **Between level** | |  |  |  |  |
|  | Intercept | -1.267 | 0.141 | -8.992 | <0.0001 |
|  | Slope_follicular phase - ovulation_ | 2.587 | 1.009 | 2.563 | 0.010 |
|  | Slope_ovulation - luteal phase_ | -1.151 | 0.970 | -1.187 | 0.235 |
|  | Testosterone_cm_ | 0.900 | 1.087 | 0.835 | 0.404 |
|  | Age | -0.001 | 0.026 | -0.029 | 0.977 |
|  | Relationship status | -0.090 | 0.268 | -0.337 | 0.736 |
|  | Socio-sexuality index | 0.188 | 0.069 | 2.739 | 0.006 |
|  |  |  |  |  |  |
| **Variance terms** | |  |  |  |  |
|  | Intercept | 0.386 | 0.126 | 3.072 | 0.002 |
|  | Slope_follicular phase - ovulation_ | 0.042 | 0.018 | 2.372 | 0.009 |
|  | Slope_ovulation - luteal phase_ | 0.019 | 0.010 | 1.917 | 0.028 |
|  |  |  |  |  |  |
| **Parameter contrasts** | |  |  |  |  |
|  | Testosterone_cm - cmc_ | 0.788 | 1.167 | 0.675 | 0.499s |

1. Marcinkowska UM, Kaminski G, Little AC, Jasienska G. Average ovarian hormone levels, rather than daily values and their fluctuations, are related to facial preferences among women. Hormones and behavior. 2018;102:114-9.
